# Distinct but Intertwined Evolutionary Histories of Multiple Salmonella enterica Subspecies

**DOI:** 10.1128/mSystems.00515-19

**Published:** 2020-01-14

**Authors:** Cooper J. Park, Cheryl P. Andam

**Affiliations:** a Department of Molecular, Cellular and Biomedical Sciences, University of New Hampshire, Durham, New Hampshire, USA; Dalhousie University

**Keywords:** *Salmonella*, genome, pan-genome, recombination, subspecies

## Abstract

*Salmonella* is responsible for many nontyphoidal foodborne infections and enteric (typhoid) fever in humans. Of the two *Salmonella* species, Salmonella enterica is highly diverse and includes 10 known subspecies and approximately 2,600 serotypes. Understanding the evolutionary processes that generate the tremendous diversity in *Salmonella* is important in reducing and controlling the incidence of disease outbreaks and the emergence of virulent strains. In this study, we aim to elucidate the impact of homologous recombination in the diversification of S. enterica subspecies. Using a data set of previously published 926 *Salmonella* genomes representing the 10 S. enterica subspecies and Salmonella bongori, we calculated a genus-wide pan-genome composed of 84,041 genes and the S. enterica pan-genome of 81,371 genes. The size of the accessory genomes varies between 12,429 genes in S. enterica subsp. *arizonae* (subsp. IIIa) to 33,257 genes in S. enterica subsp. *enterica* (subsp. I). A total of 12,136 genes in the *Salmonella* pan-genome show evidence of recombination, representing 14.44% of the pan-genome. We identified genomic hot spots of recombination that include genes associated with flagellin and the synthesis of methionine and thiamine pyrophosphate, which are known to influence host adaptation and virulence. Last, we uncovered within-species heterogeneity in rates of recombination and preferential genetic exchange between certain donor and recipient strains. Frequent but biased recombination within a bacterial species may suggest that lineages vary in their response to environmental selection pressure. Certain lineages, such as the more uncommon non-*enterica* subspecies (non-S. enterica subsp. *enterica*), may also act as a major reservoir of genetic diversity for the wider population.

**IMPORTANCE** S. enterica is a major foodborne pathogen, which can be transmitted via several distinct routes from animals and environmental sources to human hosts. Multiple subspecies and serotypes of S. enterica exhibit considerable differences in virulence, host specificity, and colonization. This study provides detailed insights into the dynamics of recombination and its contributions to S. enterica subspecies evolution. Widespread recombination within the species means that new adaptations arising in one lineage can be rapidly transferred to another lineage. We therefore predict that recombination has been an important factor in the emergence of several major disease-causing strains from diverse genomic backgrounds and their ability to adapt to disparate environments.

## Abstract (precis)

S. enterica is a major foodborne pathogen, which can be transmitted via several distinct routes from animals and environmental sources to human hosts. Multiple subspecies and serotypes of S. enterica exhibit considerable differences in virulence, host specificity, and colonization. This study provides detailed insights into the dynamics of recombination and its contributions to S. enterica subspecies evolution. Widespread recombination within the species means that new adaptations arising in one lineage can be rapidly transferred to another lineage. We therefore predict that recombination has been an important factor in the emergence of several major disease-causing strains from diverse genomic backgrounds and their ability to adapt to disparate environments.

## INTRODUCTION

*Salmonella* is widely known for causing nontyphoidal foodborne infections and enteric (typhoid) fever in humans (1–3). It is a major public health concern, causing 93.8 million illnesses and 155,000 deaths per year globally (2). Salmonellosis in humans manifests itself as diarrhea, fever, and abdominal pain within 12 to 72 h after infection (3). Aside from being able to colonize almost all warm- and cold-blooded animals (4–6), *Salmonella* is also prevalent in environmental reservoirs (7, 8). In the United States, food products such as vegetables, fruits, and meat have been identified as vehicles of *Salmonella*-associated foodborne outbreaks in the past decade (9). The emergence of antimicrobial-resistant *Salmonella* lineages further exacerbates the burden caused by this pathogen and compromises our ability to treat clinical infections (10, 11).

*Salmonella* consists of two named species, Salmonella bongori and Salmonella enterica, with the latter further classified into 10 subspecies: *enterica* (subsp. I), *salamae* (subsp. II), *arizonae* (subsp. IIIa), *diarizonae* (subsp. IIIb), *houtenae* (subsp. IV), *indica* (subsp. VI), unnamed subsp. VII, and three novel subspecies A, B, and C (12). S. enterica consists of approximately 2,600 different serotypes (13, 14), but only a few serotypes cause the majority of gastroenteritis (food poisoning) cases (2). Approximately 99% of salmonellosis is due to S. enterica subsp. *enterica* (subsp. I) serotypes, with 70% caused by only 12 serotypes (13, 14). In the United States, the two most common serotypes are S. enterica serovar Enteritidis and S. enterica serovar Typhimurium (9). S. enterica subsp. *enterica* (subsp. I) represents the vast majority of *Salmonella* strains isolated from humans and warm-blooded animals, while all the other subspecies and S. bongori are more typically isolated from cold-blooded animals (2, 15).

There is a critical need to define the processes that shape how the success of S. enterica results from the combination of intrinsic genomic factors, evolutionary processes, and the selective environment (ecology), which favors the emergence of new lineages or those with novel characteristics that enhance their resistance, virulence, or transmission. One important process that contributes to a pathogen’s success is recombination, which can rapidly spread adaptive alleles and novel genes across the population (16, 17). Hence, recombination can significantly impact the pathogen’s response to selective pressures from clinical interventions such as antibiotic use, host immune responses, and extrahost environments (18–20). Previous studies have shown that frequent recombination and the acquisition of novel genes have contributed to the ecology, evolution, and pathogenicity of S. enterica (21, 22), with evidence of recombination affecting the diversity of the lipopolysaccharide antigenic factor (23), animal host range (24), and antimicrobial resistance (10, 11). Understanding the role of recombination in *Salmonella* diversity will be particularly crucial in reducing and controlling incidence of disease outbreaks and the emergence of antimicrobial resistance in this pathogen.

In this study, we aim to compare the genomic content and elucidate the impact of homologous recombination on the diversification of the different S. enterica subspecies. Using a data set of 926 previously published *Salmonella* genomes, representing the 10 S. enterica subspecies and S. bongori, we report marked differences in core and accessory genome content between subspecies. We identified genomic hot spots of recombination that include genes associated with flagellin and the synthesis of methionine and thiamine pyrophosphate. Last, we uncovered heterogeneity and biases in rates and patterns of recombination. We interpret these findings as indicating the presence of genetic or ecological influences that facilitate the creation of hubs of gene flow between lineages and barriers between other lineages. Our results also highlight the role of the more uncommon non-*enterica* subspecies (non-S. enterica subsp. *enterica*) as a major reservoir of genetic diversity for the wider population. Our study offers important insights into within-species diversification, ecological adaptation, and cocirculation of multiple *Salmonella* lineages.

## RESULTS

### Pan-genome characteristics of *Salmonella*.

**FIG 1:**
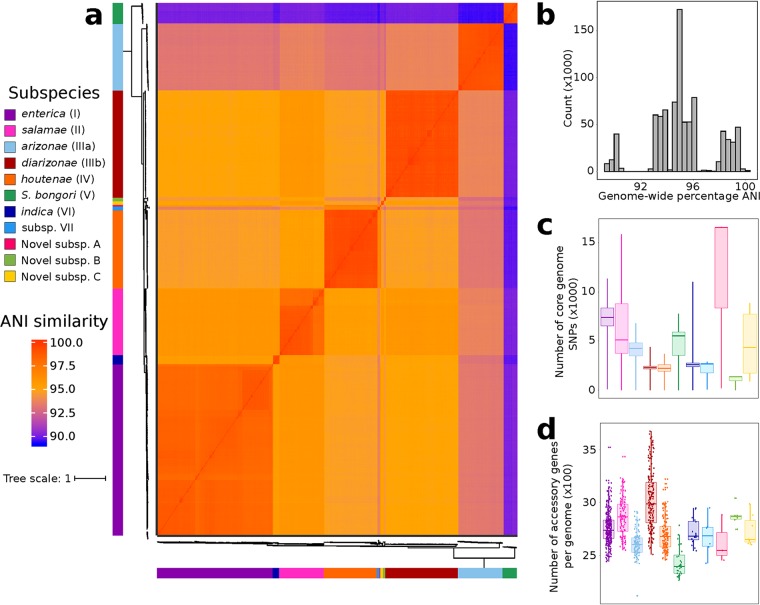
Genomic differences among Salmonella enterica subspecies genomes. (a) Pairwise genome-wide ANI values. ANI calculates the average nucleotide identity of all orthologous genes shared between any two genomes. The phylogeny was reconstructed using the concatenated alignment of 1,596 genus-wide core genes. The scale bar represents nucleotide substitutions per site. (b) Frequency distribution of all pairwise ANI values. The 95% ANI cutoff is a frequently used standard for species demarcation. (c) Number of SNPs in the core genome alignment per subspecies. The box shows the median SNP count and the lower and upper quartiles. The whiskers represent the minimum and maximum SNP counts. (d) Number of accessory genes per genome for each subspecies. Subspecies classification is based on core genome variation calculated by Alikhan et al. (12).

To investigate the relative contributions of homologous recombination to the genomic diversity of S. enterica subspecies, we compiled a total of 926 representative genomes downloaded from EnteroBase (see Table S1 in the supplemental material) (12, 25). We also included S. bongori because we hypothesized that recombination also occurs between the two species. Of the 10 S. enterica subspecies, three were reported to be novel (referred to as subspecies A, B, and C [12]) (Fig. 1a). The core genome-based phylogenetic relationships of these 926 genomes and the discovery of the novel subspecies have been published elsewhere (12). Subspecies classification in this data set was based on core single nucleotide polymorphisms (SNPs), which revealed 10 distinct S. enterica subspecies (12). Across the entire data set, genome size varied between 4.01 and 5.76 Mb (mean, 4.8 Mb) and the number of predicted genes ranged from 3,745 to 5,593 (mean, 4,564) (Table S1).

10.1128/mSystems.00515-19.1 TABLE S1 Pan-genome analyses. (a) Accession numbers and genome characteristics of *Salmonella* genomes used in this study. (b) Pan-genome analysis for *Salmonella*, S. bongori, and each S. enterica subspecies calculated by Roary. (c) List of core genes for the entire data set (genus level) identified by Roary. (d) List of core genes for S. enterica identified by Roary. Download Table S1, XLSX file, 0.1 MB. Copyright © 2020 Park and Andam. 2020 Park and Andam This content is distributed under the terms of the Creative Commons Attribution 4.0 International license.

We used Roary (26) to estimate the pan-genome of the entire *Salmonella* data set and of each subspecies. Roary classifies orthologous gene families into core genes (present in 99% ≤ strains ≤ 100%), soft core genes (present in 95% ≤ strains < 99%), shell genes (present in 15% ≤ strains < 95%), and cloud genes (present in < 15% of strains) (see Table S1 and Fig. S1 in the supplemental material). At the genus level, we found a considerably small core genome at the genus level, composed of 1,596 genes, which represents a mere 1.90% of the entire pan-genome (84,041 genes; Table S1). For S. enterica, core genes make up 2.28% (1,858 genes) of the species pan-genome (81,371 genes; Table S1). It is also notable that the vast majority of accessory genes of S. enterica (75,631 genes, representing 92.95% of the pan-genome) are present in less than 15% of the genomes, with most accessory genes also being unique to a strain (33,474 genes, representing 41.14% of the pan-genome). Comparing the five largest S. enterica subspecies (subsp. I, II, IIIa, IIIb, and IV), we found that the sizes of their core genomes are comparable, ranging from 2,636 genes in S. enterica subsp. *enterica* (subsp. I) to 3,292 genes in S. enterica subsp. *arizonae* (subsp. IIIa). However, we found major differences in the size of their accessory genomes. Combining the shell and cloud genes, the accessory genomes comprise 71.82% (12,429 genes in S. enterica subsp. *arizonae* [IIIa]) to 90.48% (33,257 genes in S. enterica subsp. *enterica* [I]) of the pan-genome of each subspecies (Table S1). A remarkable component of the accessory genome of S. enterica (31,809 genes, 40% of the accessory genome) is composed of strain-specific and ORFan genes (i.e., genes with no known homology to genes in other taxonomically or evolutionary lineages [27]), which have been recently reported to be significantly associated with pathogenicity in nine bacterial genera (28). Sequencing and annotation errors may also partly explain the large number of accessory genes in *Salmonella*.

10.1128/mSystems.00515-19.4 FIG S1 Gene frequency distributions for *Salmonella*, S. bongori, and each S. enterica subspecies calculated by Roary. The *x* axis shows the number of sampled genomes, and the *y* axis shows the number of genes shared among any number of genomes. Download FIG S1, PDF file, 0.05 MB. Copyright © 2020 Park and Andam. 2020 Park and Andam This content is distributed under the terms of the Creative Commons Attribution 4.0 International license.

To determine the degree of genomic relatedness and hence clarify the distinction among the S. enterica subspecies, we calculated the pairwise average nucleotide identity (ANI) for all possible pairs of genomes. ANI estimates the average nucleotide identity of all orthologous genes shared between any two genomes, and organisms belonging to the same species typically exhibit ≥95% ANI (29). The 10 S. enterica subspecies can be delineated based on their ANI (Fig. 1a) and can be clearly differentiated from S. bongori with a mean ANI between the two species of 89.95% (range, 89.20 to 90.53%) (Fig. 1b). Mean ANI across all pairs of S. enterica genomes is 94.68% (92.62 to 97.26%), while mean ANI within each S. enterica subspecies is 98.81% (range, 96.92 to 99.99%).

We also compared the core and accessory genomes within and among S. enterica subspecies. We first calculated the number of core SNP differences between any pair of genomes. Within S. enterica subsp. *salamae* (subsp. II), we found the greatest range of pairwise SNPs (between 3 and 15,846), while S. enterica subsp. *diarizonae* (subsp. IIIb) showed significantly less variation (between 1 and 4,386) despite it being one of the largest clusters in the study. As expected, we found considerably fewer SNPs within subspecies than between subspecies, with a maximum pairwise SNP count of 16,624 among genomes in subsp. A (Fig. 1c). Comparing the two *Salmonella* species, we obtained a mean of 66,486 core SNPs that differentiate them (range, 64,131 to 69,571 SNPs) (Fig. S2). We also compared the number of accessory genes per genome among the different subspecies. S. enterica subsp. *diarizonae* (IIIb) exhibited the highest mean as well as the greatest variability in the accessory gene content, ranging from 2,509 and 3,678 accessory genes per genome (Fig. 1d). However, pan-genome estimates are greatly influenced by the size of the data set being examined (30), and it is thus challenging to compare subspecies of different sizes.

10.1128/mSystems.00515-19.5 FIG S2 Comparison of the number of core SNPs for each pair of S. enterica subspecies, including S. bongori. The box shows the median SNP count and lower and upper quartiles. The whiskers represent the minimum and maximum SNP counts. Download FIG S2, PDF file, 0.01 MB. Copyright © 2020 Park and Andam. 2020 Park and Andam This content is distributed under the terms of the Creative Commons Attribution 4.0 International license.

### Lineage-specific rates of homologous recombination.

Within-species variation in rates of recombination has been previously reported in other bacterial pathogens, such as Streptococcus pneumoniae (31, 32) and Staphylococcus aureus (33). We therefore sought to determine whether this is also true for *Salmonella*. We compared rates of recombination among the different *Salmonella* subspecies because variable recombination rates between subspecies may reflect a differential response to environmental selection pressure and different capacities for adaptation (31). Because the numbers of genomes in the S. enterica subspecies are greatly dissimilar, ranging from 3 genomes in novel subsp. A to 297 in S. enterica subsp. *enterica* (subsp. I), we restricted our recombination analyses to the five largest subspecies. Under the null hypothesis of no recombination, we calculated the pairwise homoplasy index (PHI) statistic. We found significant evidence for the presence of recombination in S. enterica subsp. *enterica* (subsp. I), S. enterica *arizonae* (IIIa), S. enterica *diarizonae* (IIIb), and S. enterica *houtenae* (IV) (*P* value of <0.01 for each subspecies).

Next, using the program mcorr, we calculated the probability that a pair of genomes differs at one locus conditional on having differences in another locus, which defines the correlation profile (34). In the absence of recombination, the correlation profile will be constant (flat), while recombination will generate monotonically decaying correlations as a function of the distance between loci (34). This decay is due to each recombination event creating a sequentially identical fragment between the genomes of the donor and recipient; hence, a higher recombination rate results in a faster decay rate (34). The correlation profiles for each of the five subspecies exhibit a monotonic decay, with recombination rates decreasing as a function of the size of the homologous fragment (Fig. S3). Similar decaying correlation profiles have been calculated in other recombining pathogenic bacteria, such as Helicobacter pylori and Pseudomonas aeruginosa (34).

10.1128/mSystems.00515-19.6 FIG S3 Correlation profiles of the five largest S. enterica subspecies (I, II, IIIa, IIIb, and IV) calculated by mcorr. Download FIG S3, PDF file, 0.6 MB. Copyright © 2020 Park and Andam. 2020 Park and Andam This content is distributed under the terms of the Creative Commons Attribution 4.0 International license.

**FIG 2:**
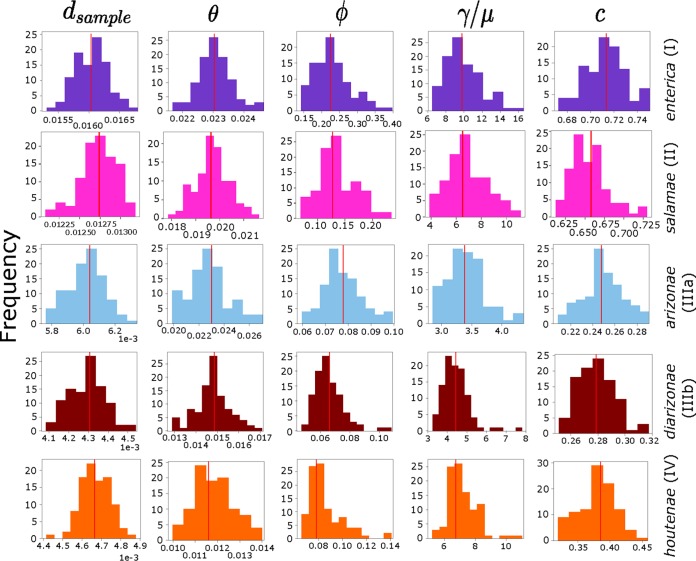
Recombination parameters of the five largest S. enterica subspecies calculated using mcorr (34). Histograms show the frequency distribution of each recombination parameter for all pairs of genomes.

We also used mcorr (34) to calculate five recombination parameters based on the correlation profiles of synonymous substitutions for pairs of homologous sequences (Fig. 2 and Table S2). As input, we used the core genes of each S. enterica subspecies and 100 bootstrap replicates. Sample diversity (*d*), which is generated from both recombination and accumulation of mutations of the clonal lineage, ranged from 4.3 × 10^−3^ in S. enterica subsp. *diarizonae* (subsp. IIIb) to 0.016 in S. enterica subsp. *enterica* (subsp. I). For comparison, other pathogenic species of *Gammaproteobacteria* exhibit a sample diversity of 3.3 × 10^−4^ (Yersinia pestis), 0.014 (P. aeruginosa), and 0.031 (Acinetobacter baumannii and Klebsiella pneumoniae) (34). The mutational divergence (θ), which refers to the mean number of mutations per locus since the divergence of a pair of homologous sites, ranged from 0.012 in S. enterica subsp. *houtenae* (subsp. IV) to 0.023 in S. enterica subsp. *enterica* (subsp. I). For comparison, the values for mutational divergence in global collections of Y. pestis, P. aeruginosa, A. baumannii, and K. pneumoniae are 0.0091, 0.027, 0.087, and 0.13, respectively (34). Recombinational divergence (ϕ) ranged from 0.066 in S. enterica subsp. *diarizonae* (IIIb) to 0.225 in S. enterica subsp. *enterica* (I). The same parameter was reported to be 0.027, 0.29, 0.11, and 0.56 in Y. pestis, P. aeruginosa, A. baumannii, and K. pneumoniae, respectively (34). The ratio ϕ/θ (or *γ/μ*), which gives the relative rate of recombination to mutation, ranged from 3.38 in S. enterica subsp. *arizonae* (IIIa) to 9.75 in S. enterica subsp. *enterica* (I). For comparison, *γ/μ* is estimated to be 3.0, 11, 4.2, and 1.3 in Y. pestis, P. aeruginosa, A. baumannii, and K. pneumoniae, respectively (34). Last, the recombination coverage (*c*), which indicates the fraction of the genome whose diversity was derived from recombination events since its last common ancestor and ranges from 0 (clonal evolution) to 1 (complete recombination) (34), ranged from 0.248 in S. enterica subsp. *arizonae* (IIIa) to 0.714 in S. enterica subsp. *enterica* (I). This parameter is reported to be 0.033 in Y. pestis, 0.52 in P. aeruginosa, 0.40 in A. baumannii, and 0.27 in K. pneumoniae (34). Comparing the five subspecies across each parameter, we found significant differences (*P* value of <0.01 for each parameter; Kruskal-Wallis test). Overall, we found that the degree in which the S. enterica subspecies differ from each other in terms of the five recombination parameters is comparable to those found when comparing different bacterial species.

10.1128/mSystems.00515-19.2 TABLE S2 Recombination analyses. (a) Recombination parameters for the five largest S. enterica subspecies (I, II, IIIa, IIIb, and IV) calculated by mcorr. (b) List of recombined genes and their gene functions identified by fastGEAR. Gene names of paralogs are identified with a number after the gene name. Download Table S2, XLSX file, 0.3 MB. Copyright © 2020 Park and Andam. 2020 Park and Andam This content is distributed under the terms of the Creative Commons Attribution 4.0 International license.

### Heterogeneity and biases in patterns of homologous recombination.

**FIG 3:**
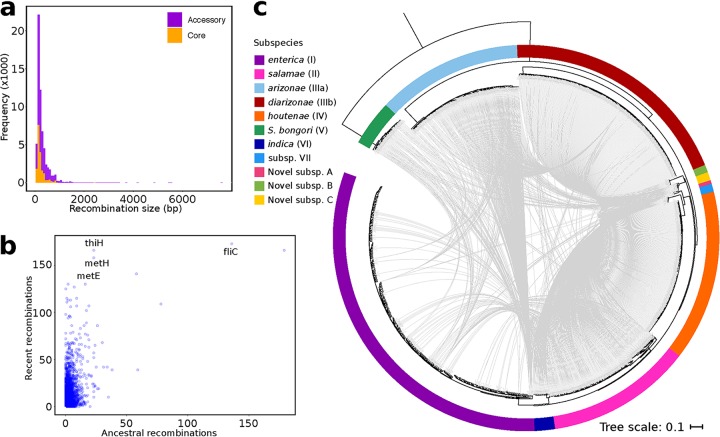
Variable patterns of recombination. (a) Size distribution of lengths of recombined core and accessory DNA fragments. (b) Genes that have undergone recent or ancestral recombination. The horizontal axis shows the estimated number of ancestral recombinations, and the vertical axis shows the estimated number of recent recombinations. For clarity, names of some of the most frequently recombined genes with known functions are shown. (c) The maximum likelihood phylogenetic tree was calculated using the concatenation of 1,596 core genes present in all 926 genomes and rooted using S. bongori. The scale bar represents nucleotide substitutions per site. The outer ring shows the different subspecies identified by Alikhan et al. (12). For visual clarity, only intersubspecies highways of recombination events identified by fastGEAR are shown (as gray arrowlines), and nonhighway recombination pairs are not shown. Inferred recipient genomes are indicated by the arrowheads.

Recent population genomic studies have reported variation not only in rates of recombination among members of a single bacterial species but also in other characteristics of recombination (31, 34, 35). One such variation can be found in the length of recombined DNA sequences. In bacterial genomes, two distinct modes of recombination have been proposed to occur: microrecombination (frequent exchange of short DNA fragments) and macrorecombination (occasional larger replacements, usually associated with major phenotypic changes) (36). To determine the size distribution of recombined DNA segments, we ran fastGEAR (37) on individual sequence alignments of core and shared accessory genes. In the entire *Salmonella* data set, the lengths of the recombination fragments greatly varied, ranging in size from 101 bp to 2,712 bp in the core genome and from 101 bp to 7,606 bp in the accessory genome (Fig. 3a). Among the five largest subspecies, the number of recombination events range from 1,604 in S. enterica subsp. *houtenae* (subsp. IV) to 5,260 in S. enterica subsp. *enterica* (subsp. I). Overall, the sizes of recombination events follow a geometric distribution, with majority of recombination events encompassing short DNA segments of <1,000 bp. Large recombination events (>1,000 bp) occurred less frequently, with the longest recombination block detected in a genome from novel subsp. A (7,606 bp). For comparison, macrorecombination in other bacterial species such as the highly recombining S. pneumoniae has been reported to reach up to 100,000 bp (32).

The strength of fastGEAR is its ability to identify both recent (affecting a few strains) and ancestral (affecting entire lineages) recombinations (37). We found that, of the 84,041 genes that comprise the *Salmonella* pan-genome, a total of 12,136 genes have had a history of recombination, representing 14.44% of the pan-genome (Fig. 3b and Table S2). Of these, 6,722 genes were involved only in recent recombination, 1,071 genes only in ancestral recombination, and 4,343 genes in both recent and ancestral recombination. Of the 12,136 recombining genes, 1,475 are core genes, and the remaining 10,661 are accessory genes. Some of the most frequently recombining genes have unknown or hypothetical functions, while those genes with the highest frequencies of recombination and which also have known functions include *fliC*, *thiH*, *metE*, and *metH* and will be highlighted here (Fig. 3b). The flagellin gene *fliC* encodes the *Salmonella* phase 1 antigen and, along with *fliB* (which encodes the phase 2 antigen), is considered a *Salmonella* serotype determinant gene (38). Flagellin genes contribute to ecological adaptation of *Salmonella* by allowing the cell to adjust their expression through phase variation when it encounters a new niche (39) and in the generation of new serotypes (40). Flagellar motility plays a role in host colonization, surface adhesion, and biofilm formation; hence, they are also important virulence factors in *Salmonella* (41). The *thiH* gene is involved in the biosynthesis of thiamine pyrophosphate, an essential cofactor for several enzymes in central metabolism and amino acid biosynthesis (42). The specific contribution of thiamine pyrophosphate in *Salmonella* pathogenicity is unclear; however, it has been reported that thiamine acquisition is a critical step in the replication and proliferation of Listeria monocytogenes within host cells during the infection process (43). The products of *metE* and *metH* are transmethylases that function in cobalamin-independent and cobalamin-dependent reactions, respectively, during the last step of methionine biosynthesis (44). While the specific role of MetE and MetH in S. enterica infection remains unclear, these genes have been reported to contribute to metabolic adaptation to physiological host conditions and pathogenicity in Ralstonia solanacearum during plant infection (45). Other recombining genes detected by fastGEAR are listed in Table S2. The phylogenies of the *metE*, *metH*, and *thiH* genes show that strains of the same subspecies often cluster together, and rarely do we find strains from one subspecies grouping within another subspecies (Fig. S4). In contrast, the *fliC* gene tree reveals numerous instances of phylogenetic incongruence, with multiple strains from one subspecies grouping with members of other subspecies. We also observed that paralogous gene families exhibit different numbers of recombination events. For example, fastGEAR identified 173, 3, 53, and 1 recent recombination events in the flagellin genes *fliC*, *fliC*_1, *fliC*_2, and *fliC*_5, respectively, and 7, 2, 67, 0, and 2 recent recombination events in the aldehyde-alcohol dehydrogenase genes *adhE*, *adhE*_1, *adhE*_2, *adhE*_3, and *adhE*_5, respectively (Table S2). We also explored evidence for recombination in the 115 plasmid-associated genes in the plasmid sequence of S. enterica subsp. *enterica* serovar Typhimurium strain LT2 genome that we used as a reference. A total of 112/753 plasmid-associated genes (i.e., 753 genes from the *Salmonella* pan-genome with an E value of 1e−10 or lower compared to any of the 115 reference plasmid genes using BLASTN) have experienced recombination (Fig. S5, Fig. S6, and Table S3). We also observed that the genes that comprise an operon do not show similar frequencies of recombination (Fig. S7 and Table S3).

10.1128/mSystems.00515-19.3 TABLE S3 Plasmid and operon analyses. (a) Presence and absence of plasmid-associated genes using the plasmid sequence of S. enterica subsp. *enterica* serovar Typhimurium strain LT2 as a reference. (b) List of genes in S. enterica operons with detected recombination. Download Table S3, XLSX file, 0.4 MB. Copyright © 2020 Park and Andam. 2020 Park and Andam This content is distributed under the terms of the Creative Commons Attribution 4.0 International license.

10.1128/mSystems.00515-19.7 FIG S4 Maximum likelihood gene trees of *fliC, thiH, metH*, and *metE* built using RAxML using a general time-reversible (GTR) nucleotide substitution model, four gamma categories for rate heterogeneity, and 100 bootstrap replicates. The scale bar represents nucleotide substitutions per site. Red circles mark branches with at least 60% bootstrap support. Gene trees for *metE and metH* were rooted using S. bongori as an outgroup. The *fliC* and *thiH* gene trees were unrooted because the genes were not detected in S. bongori. The colored bars show subspecies (left) and BAPS lineage (right). Download FIG S4, PDF file, 0.2 MB. Copyright © 2020 Park and Andam. 2020 Park and Andam This content is distributed under the terms of the Creative Commons Attribution 4.0 International license.

10.1128/mSystems.00515-19.8 FIG S5 Presence or absence of plasmid-associated genes in the plasmid sequence of S. enterica subsp. *enterica* serovar Typhimurium strain LT2 used as a reference. The phylogenetic tree is identical to the tree in Fig. 1a and 3c. Green blocks indicate the presence of a gene in syntactic order with the first plasmid gene at the top. The plasmid genes are listed in Table S3 in the same order as shown in this figure. Download FIG S5, PDF file, 0.10 MB. Copyright © 2020 Park and Andam. 2020 Park and Andam This content is distributed under the terms of the Creative Commons Attribution 4.0 International license.

10.1128/mSystems.00515-19.9 FIG S6 Recent and ancestral recombination in plasmid-associated genes. The plasmid genes and their recombination frequencies are listed in Table S3. Download FIG S6, PDF file, 0.03 MB. Copyright © 2020 Park and Andam. 2020 Park and Andam This content is distributed under the terms of the Creative Commons Attribution 4.0 International license.

10.1128/mSystems.00515-19.10 FIG S7 Frequency of recent recombination of individual genes of an operon. Each alternating column of dots (purple and orange) along the *x* axis denotes an operon, with each point in the column identifying a gene. The *y* axis represents the frequency of recent recombination events identified by fastGEAR. Download FIG S7, PDF file, 0.1 MB. Copyright © 2020 Park and Andam. 2020 Park and Andam This content is distributed under the terms of the Creative Commons Attribution 4.0 International license.

Highways of recombination, whereby a pair of strains or lineages frequently recombine with each other more often than they do with others, have been previously reported in the Gram-positive S. pneumoniae (31). Here, we aim to determine whether such highways of recombination also exist in *Salmonella*. To achieve this, we first identified the recombining pairs of donor and recipient genomes. Using the method developed in the S. pneumoniae study (31), we first calculated the sum of a potential donor’s probability score across every recombination event in every gene as its probability of being a recombination donor. We then assigned the role of the most probable donor in each recombination event to the genome with the highest cumulative donor probability score. For each pair, we characterized it as one linked by a highway of recombination when the number of recombination events from donor to recipient was at least 1 standard deviation above the average number of recombination events per recombining pair across the entire data set. We also considered the direction of recombination events, which means that any pair of recombining genomes can be linked by a highway in either direction. We identified a total of 38,105 unique recombining pairs of genomes in the entire *Salmonella* data set, of which 2,190 fit our definition of a highway. Of these, a total of 1,784 are highways that linked genomes from different subspecies (Fig. 3c). Last, we also found that 86% of strains in the data set acted as a DNA donor, while every genome has received recombined DNA at least once.

## DISCUSSION

S. enterica continues to threaten animal and human health worldwide. While S. enterica subsp. *enterica* (subsp. I) accounts for the majority of clinical infections, little is known of how other subspecies contribute to the virulence and adaptive potential of the entire species. To elucidate its success as a pathogen, analyses of the genomic structure and phylogenetic relationships among the different S. enterica subspecies is critical. Here, we show that recombination within and between subspecies has played a major role in shaping the evolution and genome structure of S. enterica. Widespread recombination within the species means that new adaptations arising in one lineage can be rapidly transferred to another distantly related lineage (16, 17).

The major finding in this study is that while the different S. enterica subspecies can be distinguished from each other based on their core and accessory genomes, variation in recombination frequencies occurs between the different subspecies. Our findings greatly expand on the results of a previous study that reported an uneven role of recombination among S. enterica subsp. *enterica* (subsp. I) lineages based on sequencing approximately 10% of their core genome (21). In that study, the authors reported that some lineages displayed evidence of more frequent recombination than others and that recombination has occurred predominantly between members of the same lineage, thus suggesting barriers to recombination (21). More recently, a recombination analysis of 73 S. enterica genomes using coancestry and hybridization methods also show variation in recombination across the species, resulting in the formation of hybrid groups within the genus (46). Variability in gene content and in patterns of recombination may be considered effective strategies for a species to maintain potentially useful adaptive alleles and novel genes that can be rapidly disseminated with other members of the species. This variation may also prevent the complete loss of a gene from the species gene pool by retaining a copy of it in one or few strains. Within-species differences in recombination also suggest that lineages within a species respond to selective pressures and environmental changes in different ways (31). Our results also imply that recombinations are not random events that impact all members of a species in a uniform manner. Genetic or ecological influences likely exist that facilitate the creation of hubs of gene flow between certain lineages as well as barriers between other lineages. We interpret these findings as indicating the existence of both biases and barriers of recombination between multiple lineages, which can shape the phylogenetic distribution of different genetic elements independent of the organisms that harbor them (47).

Several factors can potentially explain within-species variation in the rates of recombination and biases in donor-recipient linkages. First, minimal niche overlap can impact opportunities for recombination between strains and subspecies. Non*-enterica* subspecies are often sampled from cold-blooded animals (e.g., turtles, snakes, lizards, crocodiles), while S. enterica subsp. *enterica* (subsp. I) is frequently found in humans and warm-blooded animals consumed by humans (i.e., poultry, cattle, and pigs) (15). Such ecological barriers may explain the fewer highways of recombination observed between S. enterica subsp. *enterica* (I) and the non*-enterica* subspecies compared to recombination between the different non*-enterica* subspecies. However, S. enterica subsp. *enterica* (I) and the non*-enterica* subspecies are not exclusively isolated from each other, and both can sometimes be found together in cold- and warm-blooded animals. Hence, another possible explanation for the variation in recombination is that different *Salmonella* subspecies occupy distinct microecological niches (48), which may even be separated by a few millimeters, within a human or animal host and therefore reduce the opportunity for genetic exchange. The existence of cryptic niches and their role in structuring bacterial populations have been previously reported. Two generalist Campylobacter jejuni lineages inhabiting the same animal host show no evidence of recombination between them even though they freely recombine with other lineages and with each other in the laboratory setting (49).

Certain genomic elements can also influence the success of a recombination event, thus contributing to the biases and barriers to recombination. One example is the functional linkage of multiple genes in operons. Functional similarity, and in some cases dependency, of operon-linked genes may likely limit the potential for recombination to impact individual genes in a region under positive selection and hence promote the horizontal gene transfer (HGT) of entire operons (50–52). However, it has been reported that a remarkable 35% of operons that show evidence of HGT are made up of genes with different phylogenetic affinities, occurring through *in situ* xenologous displacement through recombination (51), and thus may partly explain our result of differential recombination within an operon. Frequent homologous replacement of genes within an operon allows the bacterium to maintain operon integrity (i.e., without causing disruption of operon organization and function) in the face of strong positive selection (51). Plasmids and other mobile elements can also facilitate and influence patterns of recombination and virulence in enteric pathogens (53). In *Salmonella*, only a small number of recombining genes are associated with plasmids; hence, other mechanisms of recombination likely play a more substantial role. Future work should therefore explore the contributions of a variety of mechanisms (transduction, transformation, conjugation, other types of mobile genetic elements) in mobilizing different components of the *Salmonella* pan-genome. Additionally, incompatible restriction-modification (R-M) systems act as genetic barriers that can limit extensive recombination and incorporation of longer DNA segments (54). A previous study of S. enterica subsp. *enterica* (subsp. I) showed mosaicism in the *mutS* gene, which encodes a key component of the methyl-directed mismatch repair (MMR) system, with mutant alleles in *mutS* able to enhance the recombination between lineages (55, 56). It is possible that minute R-M differences and MMR defects can facilitate frequent recombination between certain subspecies but not with others. Future work focusing on *in vitro* recombination assays of strains from different S. enterica subspecies may provide important insights into whether genetic, mechanistic, or ecological barriers can explain biases in recombination partners.

The major limitation in this study is the high variability in the number of genomes in each of the 10 subspecies, making it difficult to elucidate and compare the novel but less well-known subspecies with the more prevalent S. enterica subsp. *enterica* (subsp. I). The non*-enterica* subspecies have been less studied, mainly because they are often associated with cold-blooded animals (57, 58), and cases of human salmonellosis are almost entirely limited to serotypes of S. enterica subsp. *enterica* (subsp. I) (2, 22). To date, there is therefore a stark gap in sampling and genome sequencing work that has been done on non*-enterica* subspecies. Previous reports indicate that non*-enterica* subspecies have lower invasive capacity, virulence, and levels of resistance to common antibiotics, and human infections have been mostly those involving weakened immune systems (15, 59). However, as we have shown in this study, there is frequent recombination between subspecies, hence these less well-known subspecies likely act as reservoirs of novel allelic variants or genes that human-associated lineages can sample from when needed (e.g., as a response to environmental change or host immune system). Future genome sequencing endeavors may shed important insights on the genomic diversity on many non-*enterica* subspecies from various hosts and habitats. Last, the draft nature of these genomes, potential sequencing errors, and misannotation may also have influenced our analysis of genome structure, including the characterization of core and accessory genes, detection of recombination events, and identification of donors and recipients.

Recombination, either through homologous or illegitimate means, plays a fundamental role in the evolution and species diversification of bacterial genomes (16, 60, 61). For many bacterial pathogens, including *Salmonella*, recombination has been implicated in the emergence of highly virulent lineages (10, 62, 63). Our results provide crucial insights into the contributions of recombination into the diversification and adaptive capabilities of S. enterica as a species. Understanding the extent of genomic variation within a species, and the ecological and evolutionary underpinnings of this variation, will enable successful surveillance of emerging infectious agents. It will also facilitate the development of effective clinical interventions to limit the emergence of new pathogenic clones and of accurate predictions of how specific lineages will respond to environmental changes.

## MATERIALS AND METHODS

### Data set.

Our data set consisted of 926 Salmonella enterica genomes downloaded from EnteroBase (12, 25). It consists of 297 genomes of S. enterica subsp. *enterica* (subsp. I), 116 S. enterica subsp. *salamae* (subsp. II), 116 S. enterica subsp. *arizonae* (subsp. IIIa), 187 S. enterica subsp. *diarizonae* (IIIb), 136 S. enterica subsp. *houtenae* (IV), 36 S. bongori (V), 16 S. enterica subsp. *indica* (VI), six S. enterica subsp. VII, three S. enterica subsp. A, six S. enterica subsp. B, and seven S. enterica subsp. C genomes. Classification of the genomes into subspecies was based on delineation of the core SNPs reported by Alikhan et al. (12). To maintain consistency in gene annotations, all genomes were reannotated using Prokka v1.12 (64) with default parameters.

### Pan-genome analyses.

To determine the degree of genomic relatedness and clarify the relationships between the subspecies, we calculated the genome-wide average nucleotide identity (ANI) for all possible pairs of genomes using the program FastANI v.1.0 (29). ANI is a robust similarity metric that has been widely used to resolve inter- and intrastrain relatedness. The threshold value of 95% has been often used as a cutoff for comparisons belonging to the same or different species (29). We used Roary v3.11 with default parameters (95% identity and 99% presence for core genome inclusion) (26) to characterize the pan-genome at the genus, species, and subspecies levels. Roary classifies genes into core, soft core, shell, and cloud genes by iteratively preclustering protein sequences using CD-HIT (65), all-against-all BLASTP (66), and Markov clustering (67). A strength of Roary is that it treats paralogous genes as independent gene families and splits the paralogs into separate clusters by examining the synteny (i.e., the physical colocalization of genes) of flanking genes. We used this clustering output in all downstream analyses, including the pan-genome characterization and recombination detection. Visualization of the pan-genome was done using the postprocessing scripts provided by Roary. Gene functions were inferred using the Gene Ontology Consortium’s Enrichment Analysis (68, 69). For the plasmid analysis, we downloaded the S. enterica subsp. *enterica* serovar Typhimurium strain LT2 genome and its plasmid sequence from the NCBI RefSeq database (accession identifier [ID] GCF_000006945.2) to be used as a reference. Plasmid-associated genes were identified by using BLASTN (66) to compare genes in the reference plasmid against all genes in the *Salmonella* pan-genome with a conservative E-value threshold of 1e−10. Operons were identified by running the S. enterica reference genome through the Operon-mapper web-based pipeline (70).

### Phylogeny reconstruction.

Nucleotide sequences of each single-copy orthologous gene family obtained from Roary were aligned using MAFFT v.7.305b (71). Sequence alignments of core genes were concatenated to give a single core alignment and a maximum likelihood phylogeny was then generated using the program Randomized Axelerated Maximum Likelihood (RAxML) v.8.2.11 (72) with a general time-reversible (GTR) nucleotide substitution model (73), four gamma categories for rate heterogeneity, and 100 bootstrap replicates. All phylogenies were visualized using the Interactive Tree of Life (74). Pairwise SNP differences in the core genome alignment were identified using the R script available at https://github.com/MDU-PHL/pairwise_snp_differences.

### Detection of homologous recombination.

Using the core genome alignments, we also calculated the pairwise homoplasy index (PHI) test to determine the statistical likelihood of recombination being present in the entire data set and within each subspecies (75). This statistic measures the genealogical correlation or similarity of adjacent sites. Under the null hypothesis of no recombination, the genealogical correlation of adjacent sites is invariant to permutations of the sites, as all sites have the same history (75). Significance of the observed PHI was estimated using a permutation test.

To calculate and compare rates of recombination between subspecies, we ran mcorr, which uses a coalescent-based model of evolution to calculate the probability that a pair of genomes differs at one locus conditional on having differences at another locus (34). For input for mcorr, we used the core genes identified by Roary (26) of each subspecies. The recombination parameters estimated by mcorr include θ (the average number of mutations per locus), ϕ (the average number of recombinations per locus), the ϕ/θ ratio (the number of recombination events per mutation in a population, which is comparable to *γ/μ*), *d* (the amount of diversity in a sample brought on by the effects of both recombination and clonal evolution), and *c* (the fraction of the sample diversity derived from recombination).

To identify the most frequently recombining genes across the genomes, we used fastGEAR (37) with default parameters on individual core and shared accessory genes identified by Roary. The program fastGEAR predicts recombination events by first clustering sequences into lineages using a hidden Markov model implemented in BAPS (76). These lineages are defined as groups that are genetically divergent by at least 50% of the sequence alignment. Within each lineage, each genome was then examined using a hidden Markov model that iteratively compares polymorphic sites in the strain’s sequence (relative to other members of its own lineage) against the same nucleotide site in other lineages. The comparison is made over multiple iterations of the model, each with updated parameters from the prior run. At the conclusion of the simulation, if a nucleotide site of a strain is found to be more similar to the same site in strains of another lineage, it is considered to be a recombination event. To test the significance of these inferred recombinations and identify false-positive results, fastGEAR uses a diversity test that compares the diversity of the recombined fragment in question to its background. Recombinations were visualized using R (77) and the postprocessing scripts provided by fastGEAR.

For every recent recombination event identified by fastGEAR, we inferred its donor strain by extracting the nucleotide sequence of the predicted recombined fragment and used it as a query in a BLASTN (66) search against all possible genomes from the identified donor lineage, following the methodology used to identify recombination donors in S. pneumoniae (31). The top BLAST hit with the highest bit score was considered the potential donor and given a probability score of 1 for that event, provided that it had an E value of at least 10^−10^ and at least 95% nucleotide identity. The E value and nucleotide identity values were chosen to maintain a strict conservative relationship between the donor and recipient. Following a recent recombination event, we expect that the nucleotide similarity between donor and recipient will be remarkably high, and in many cases identical. While our chosen threshold values were arbitrary from a biological perspective, they were chosen to reflect that expectation. In the event of a tie where the E value and nucleotide identity values were the same across multiple donors, the probability score for that event was divided evenly among each donor (i.e., a probability score of 0.25 was assigned in a four-way tie). This approach involves calculating the sum of a potential donor’s probability score across every recombination event in every gene as its likelihood of being a recombination donor. We then assigned the role of the most probable donor in each recombination event to the strain with the highest cumulative donor probability score. Events with potential donors of equal cumulative scores were considered to have originated from the most recent common ancestor of the donors and were discarded from the analysis as an ancestral recombination event.

### Data availability.

The genomes analyzed in this study were downloaded from and are available in the EnteroBase database (https://enterobase.warwick.ac.uk/species/index/senterica) (25). Accession numbers are listed in Table S1 in the supplemental material.
