# Supplementary material for: Distinct but Intertwined Evolutionary Histories of Multiple Salmonella enterica Subspecies
Source: mSystems. 2020 Jan 14;5(1):e00515-19. doi: 10.1128/mSystems.00515-19 (PMC6967386; doi:10.1128/mSystems.00515-19)
Supplement: FIG S1 [file mSystems.00515-19-sf001.pdf]

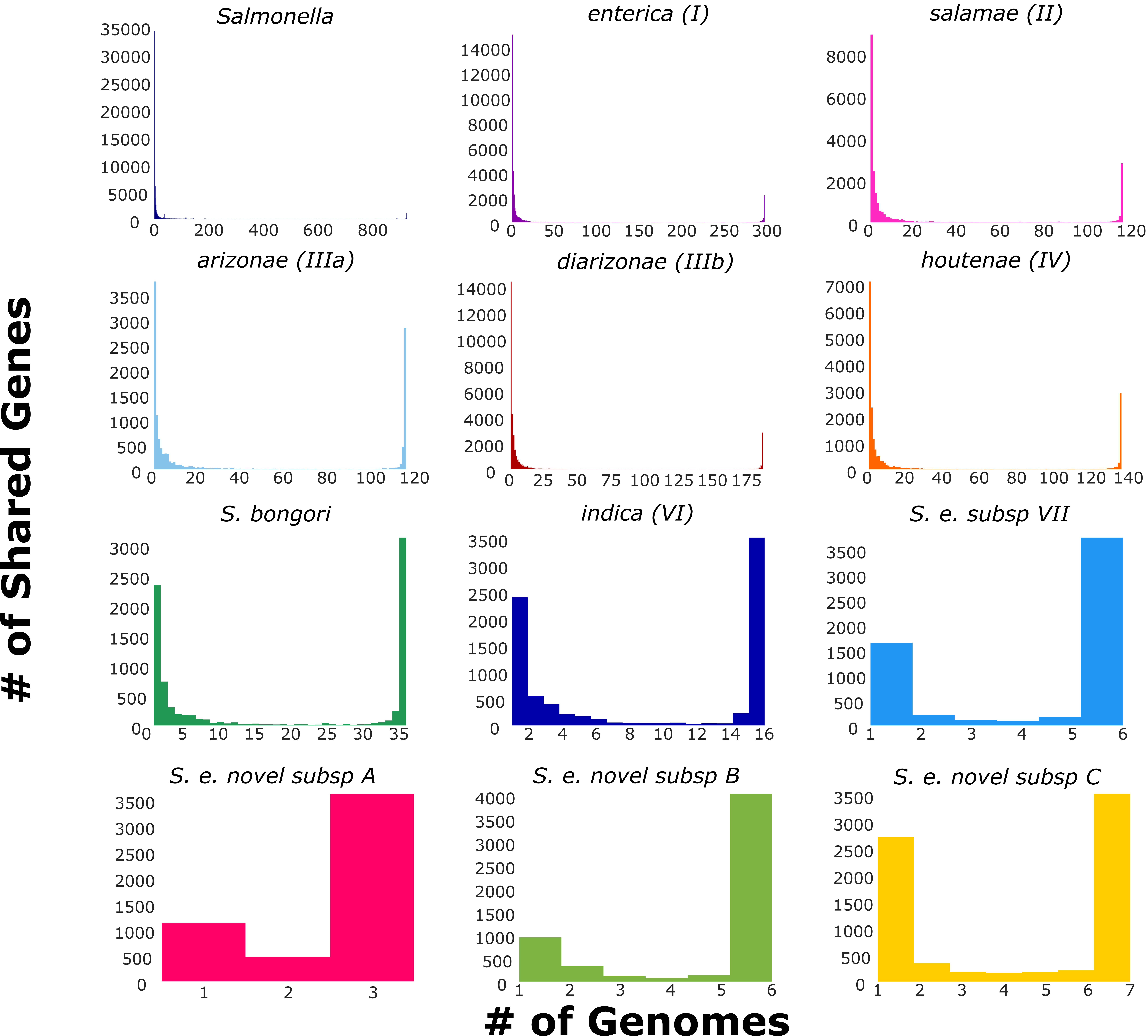

Salmonella
enterica (I)
salamae (II)
arizonae (IIIa)
diarizonae (IIIb)
houtenae (IV)
S. bongori
indica (VI)
S. e. subsp VII
S. e. novel subsp A
S. e. novel subsp B
S. e. novel subsp C
# of Shared Genes
# of Genomes
