# Supplementary figures and images for: Distinct but Intertwined Evolutionary Histories of Multiple Salmonella enterica Subspecies
Source: mSystems. 2020 Jan 14;5(1):e00515-19. doi: 10.1128/mSystems.00515-19 (PMC6967386; doi:10.1128/mSystems.00515-19)

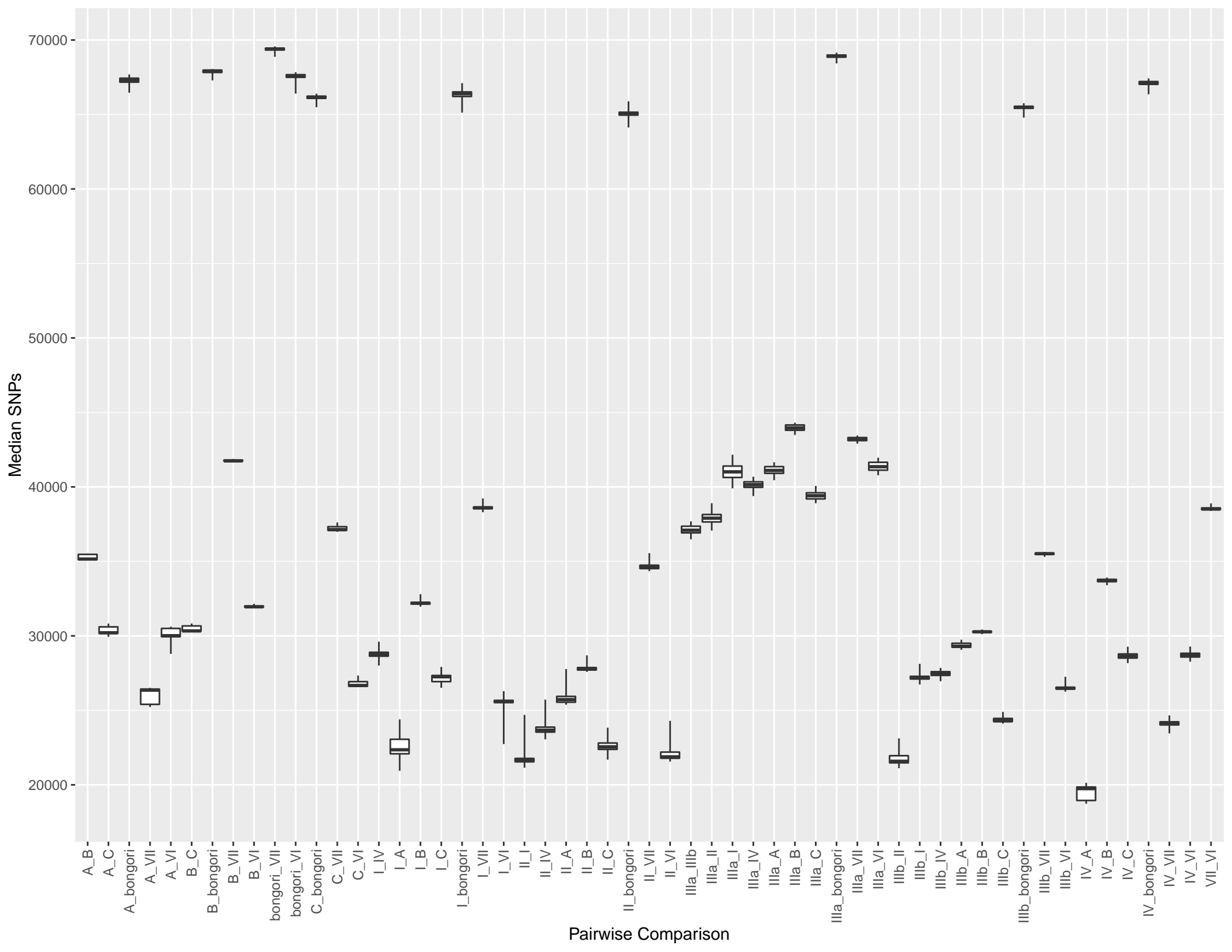

Supplement: FIG S2 [file mSystems.00515-19-sf002.pdf]

*S. e. enterica* (I)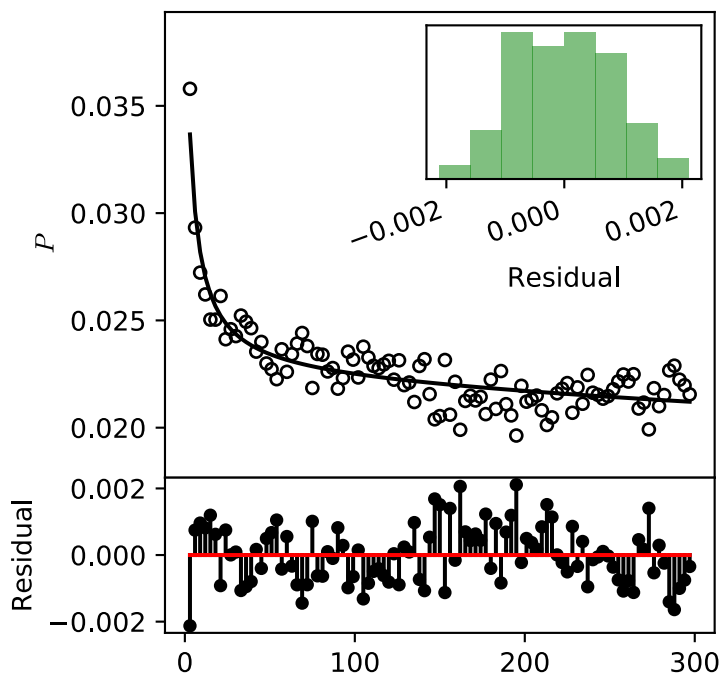*S. e. salamae* (II)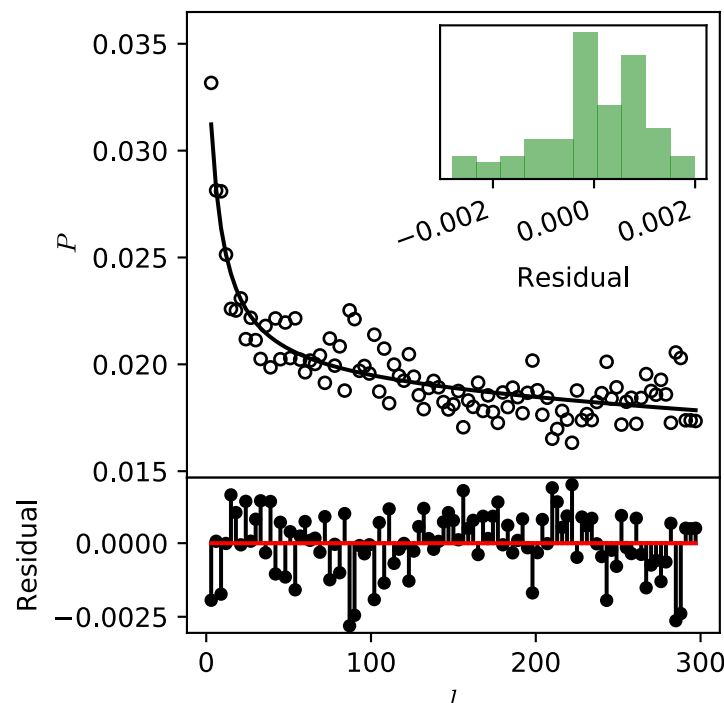*S. e. arizonae* (IIIa)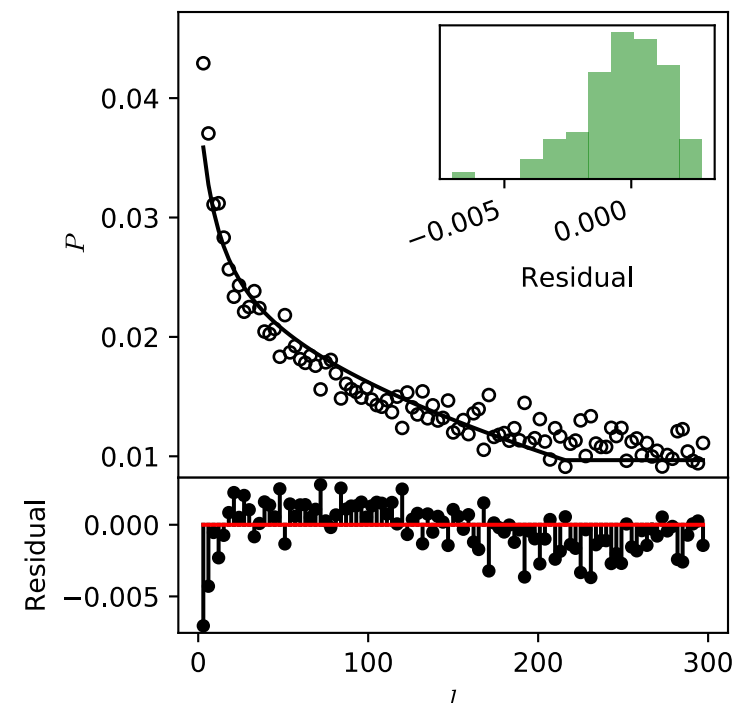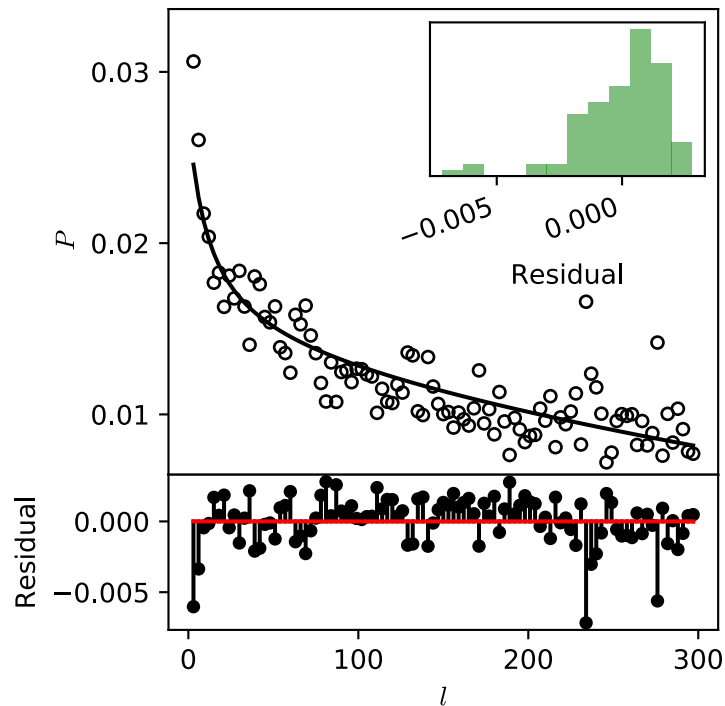*S. e. diarizonae* (IIIb)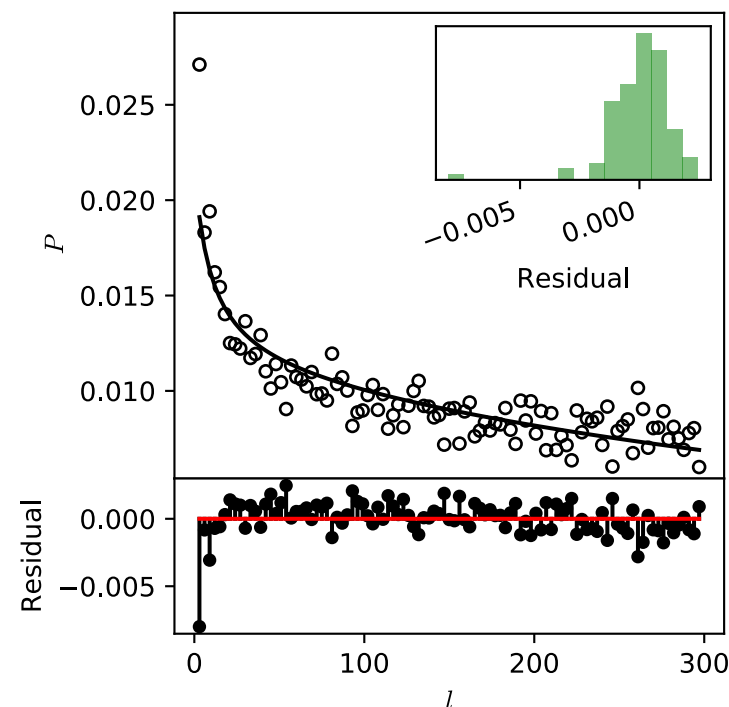*S. e. houtenae* (IV)

Supplement: FIG S3 [file mSystems.00515-19-sf003.pdf]

*fliC*

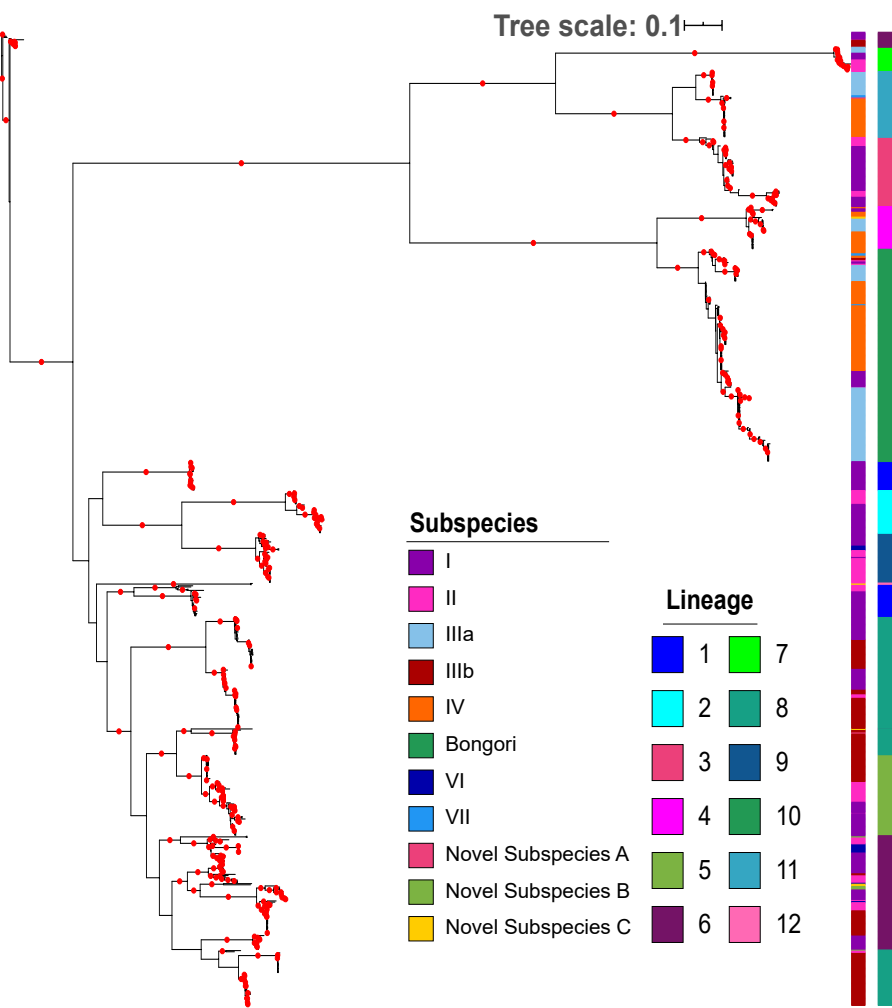

*metE*

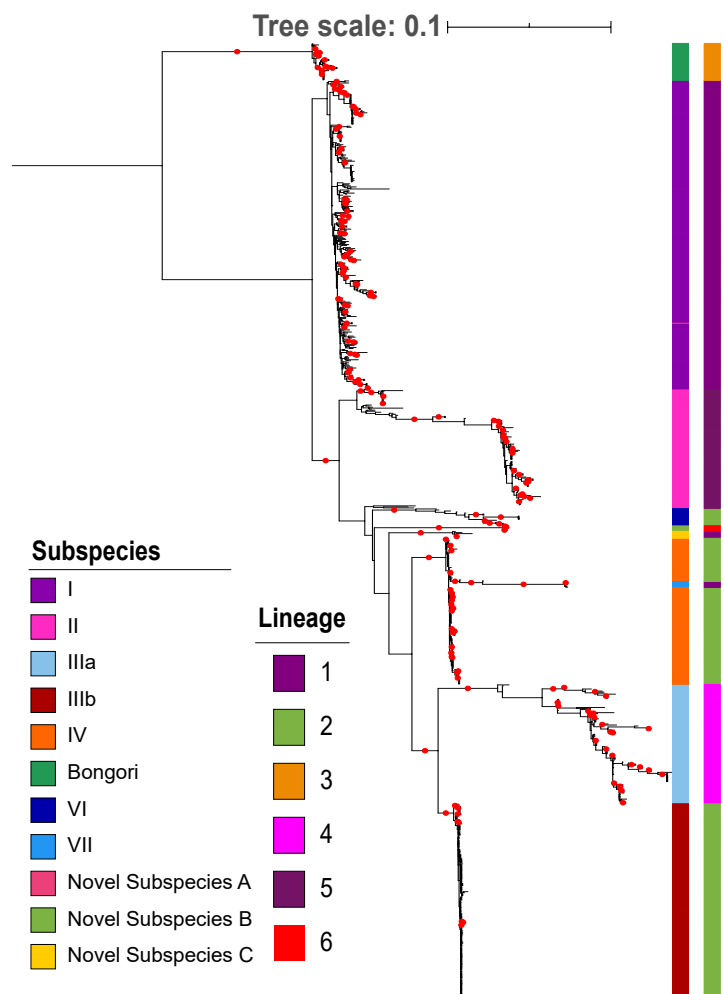

*thiH*

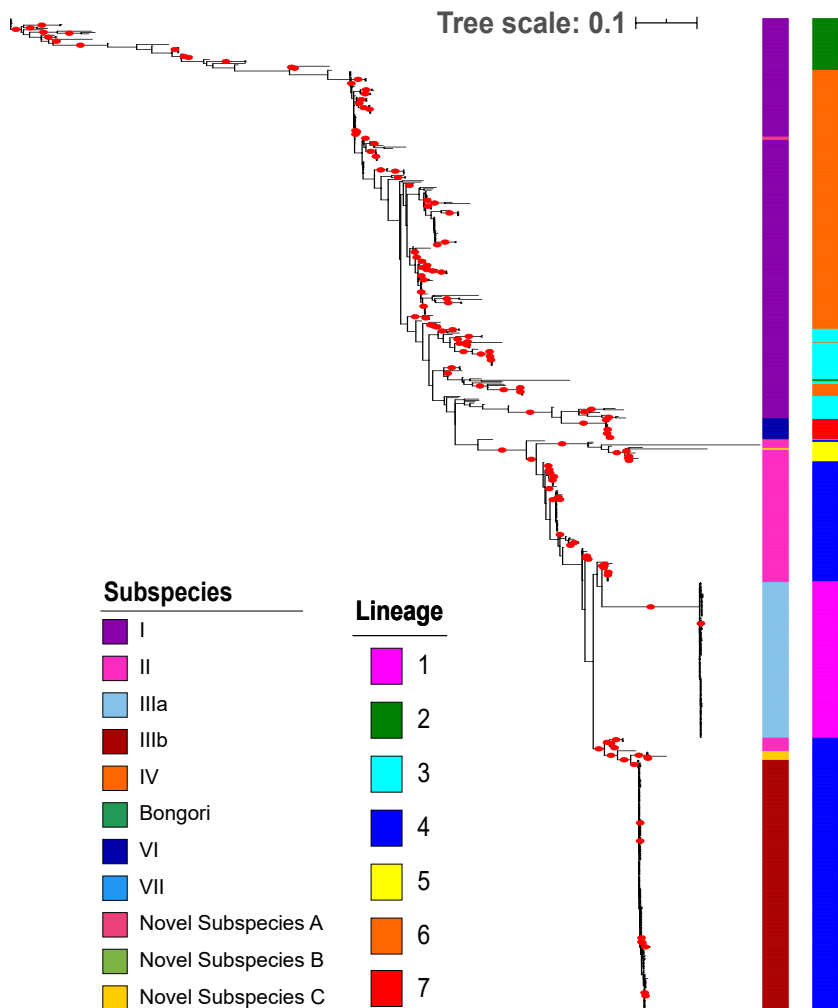

*methH*

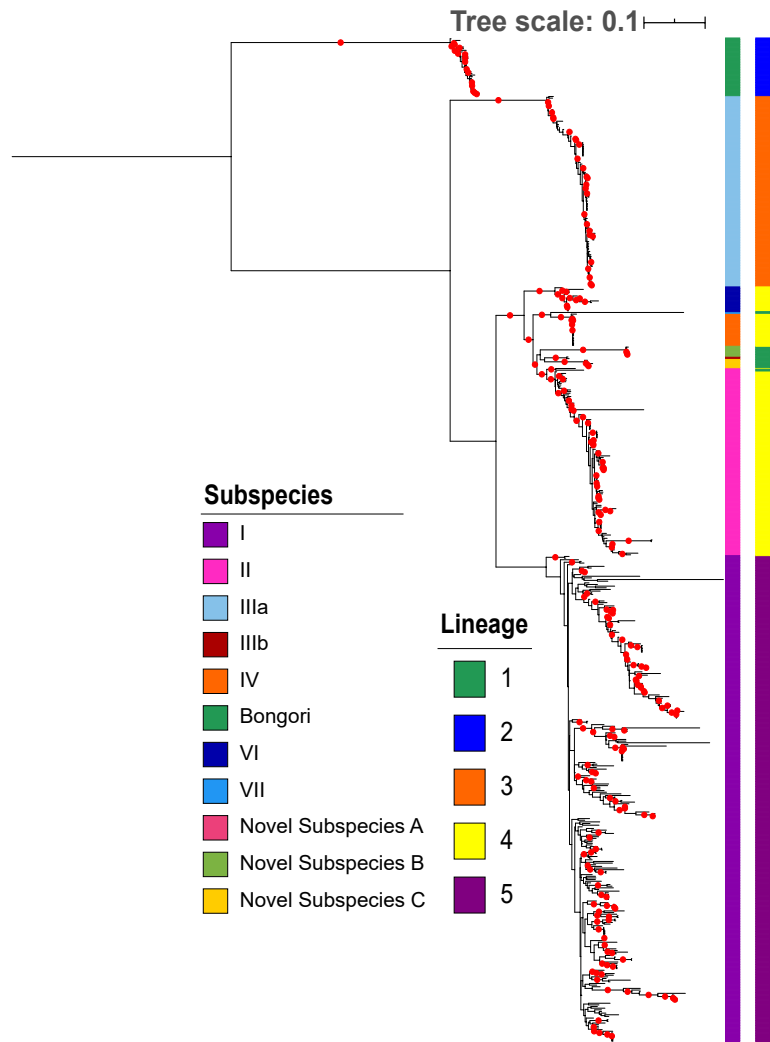

Supplement: FIG S4 [file mSystems.00515-19-sf004.pdf]

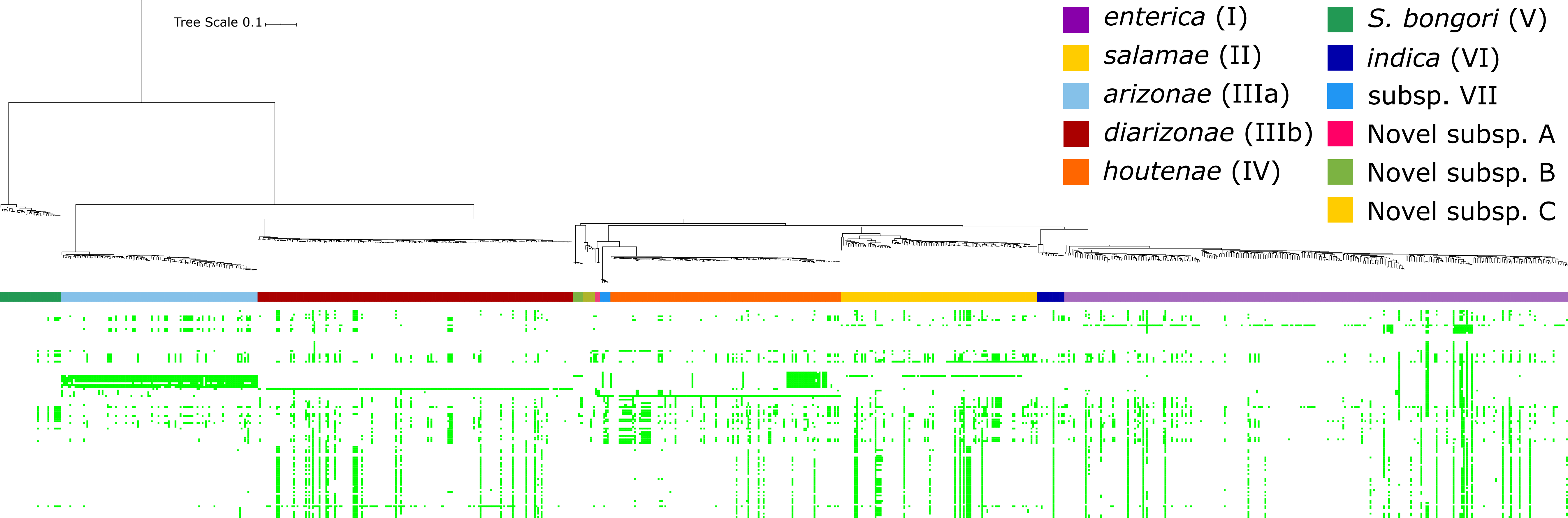

Supplement: FIG S5 [file mSystems.00515-19-sf005.pdf]

fastGEAR Ancestral vs Recent Recombinations

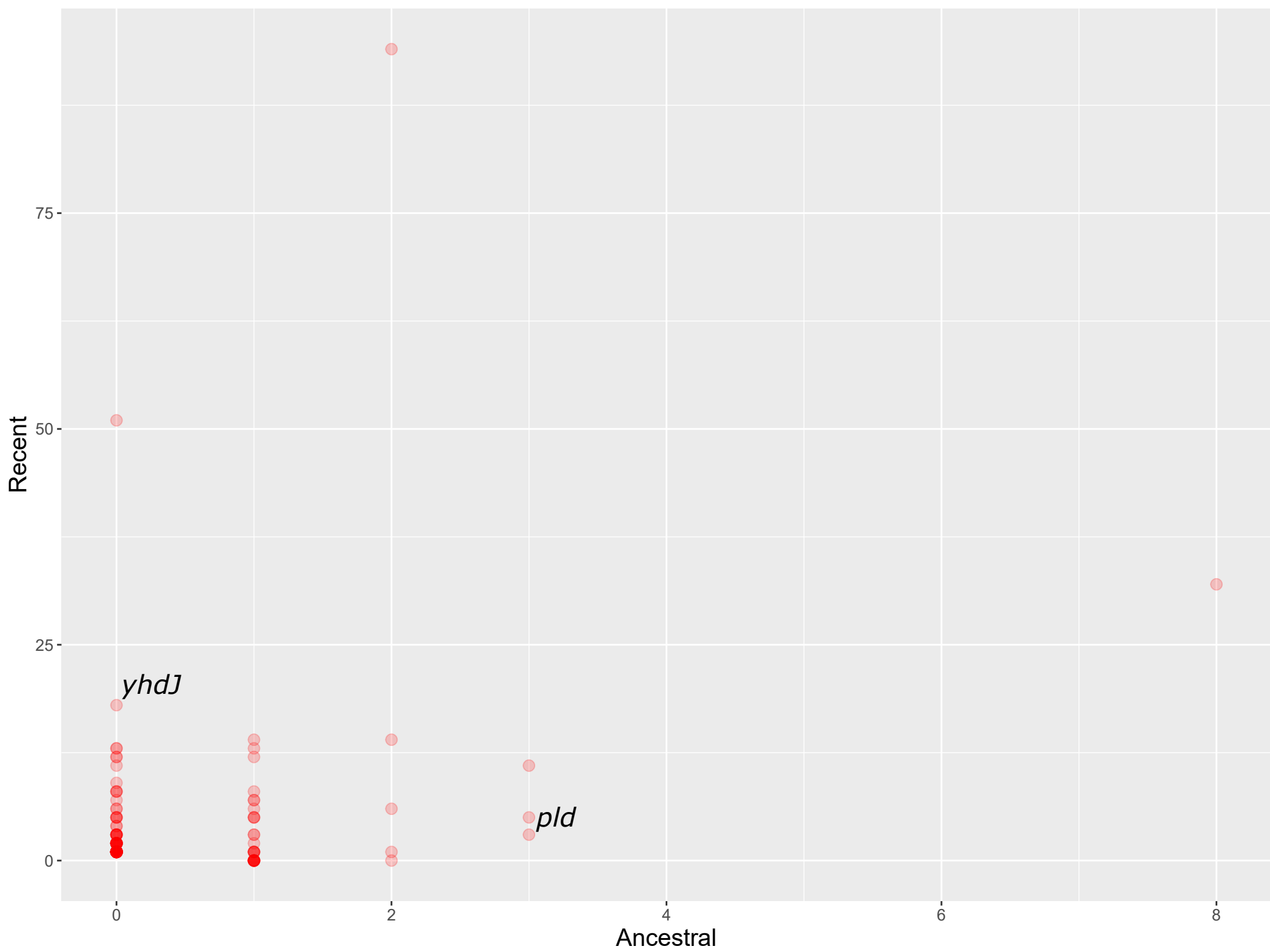

Supplement: FIG S6 [file mSystems.00515-19-sf006.pdf]

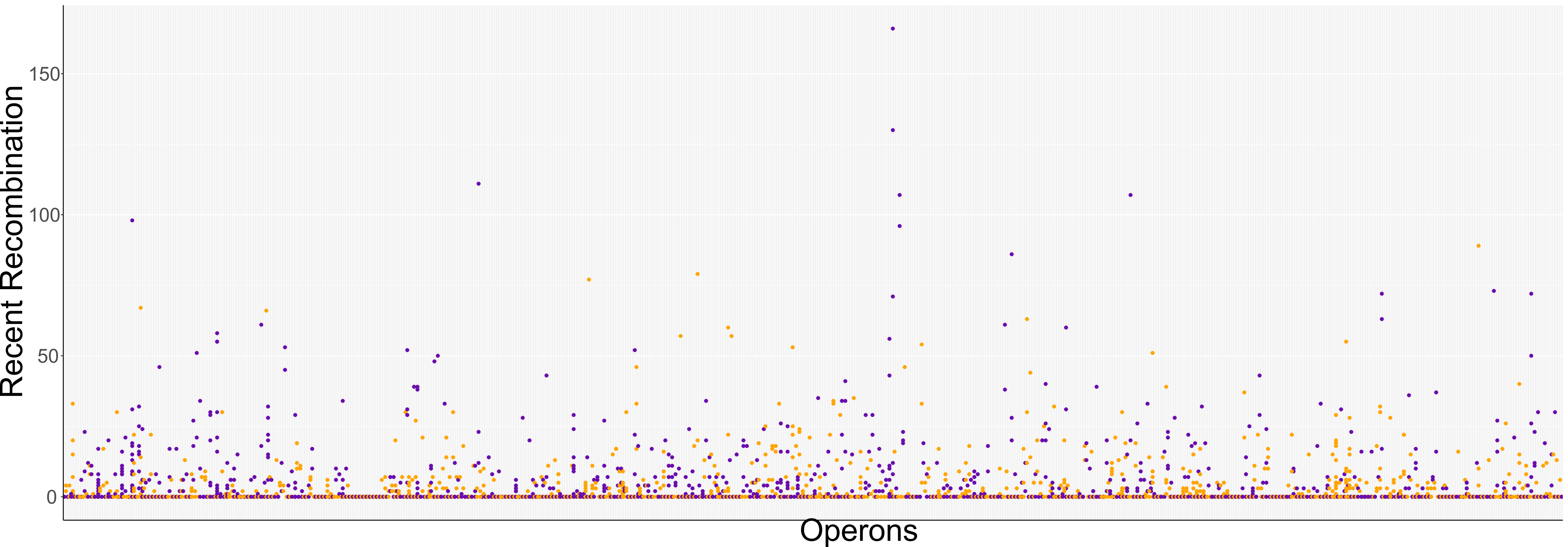

Supplement: FIG S7 [file mSystems.00515-19-sf007.pdf]
